# Aquatic Foods and Nutrition in the Pacific

**DOI:** 10.3390/nu12123705

**Published:** 2020-11-30

**Authors:** Anna K. Farmery, Jessica M. Scott, Tom D. Brewer, Hampus Eriksson, Dirk J. Steenbergen, Joelle Albert, Jacob Raubani, Jillian Tutuo, Michael K. Sharp, Neil L. Andrew

**Affiliations:** 1 Australian National Centre for Ocean Resource and Security, Faculty of Business and Law, University of Wollongong, Wollongong 2522, Australia; scottj@uow.edu.au (J.M.S.); tbrewer@uow.edu.au (T.D.B.); H.Eriksson@cgiar.org (H.E.); dirks@uow.edu.au (D.J.S.); nandrew@uow.edu.au (N.L.A.); 2 WorldFish, Honiara, Faculty of Agriculture, Fisheries and Forestry, C/O Solomon Islands National University, Ranadi, Solomon Islands; J.Wate@cgiar.org; 3 Island Elements, Brisbane 4069, Australia; joellealbert099@gmail.com; 4 Fisheries, Aquaculture and Marine Ecosystems Division, The Pacific Community, Noumea Cedex 98849, New Caledonia; jasonr@spc.int; 5 Statistics for Development Division, The Pacific Community, Noumea Cedex 98849, New Caledonia; michaels@spc.int

**Keywords:** fish consumption, malnutrition, Melanesia, dietary diversity, healthy diets, food systems, seafood trade

## Abstract

National rates of aquatic food consumption in Pacific Island Countries and Territories are among the highest in the world, yet the region is suffering from extensive levels of diet-related ill health. The aim of this paper is to examine the variation in consumption patterns and in nutrient composition of aquatic foods in the Pacific, to help improve understanding of their contribution to food and nutrition security. For this examination we analysed nutrient composition data and trade data from two novel region-specific databases, as well as consumption data from national and village level surveys for two Melanesian case studies, Vanuatu and Solomon Islands. Results demonstrated that consumption depends on availability and the amount and type of aquatic food consumed, and its contribution to nutrition security varies within different geographic and socio-demographic contexts. More data is needed on locally relevant species and consumption patterns, to better inform dietary guidelines and improve public health both now and into the future. Advice on aquatic food consumption must consider the nutrient composition and quantity of products consumed, as well as accessibility through local food systems, to ensure they contribute to diverse and healthy diets.

## 1. Introduction

Consumption rates of aquatic food(aquatic animals and plants grown in, or wild-harvested from, water and used for food or feed) in Pacific Island Countries and Territories (PICTs) are among the highest in the world [1]. Annual average consumption by coastal rural populations ranges from 30–118 kg per person in Melanesia, 62–115 kg in Micronesia, and 50–146 kg in Polynesia [2]. Aquatic food provides 50–90% of the dietary animal protein in rural areas [1]. While consumption in urban centres is lower than in rural areas, it usually still exceeds the global average of around 20 kg per person per year [3]. Aquatic foods are highly nutritious [4], however, despite the high rate of aquatic food consumption, many PICTs experience extensive levels of diet-related ill health [5].

Malnutrition, as distinct from food insecurity, is a persistent challenge in the Pacific Island region. While micronutrient deficiencies and undernutrition are major problems [6,7,8], many PICTs (PICTs include: Commonwealth of the Northern Mariana Islands *, Guam *, Palau, the Federated States of Micronesia, Marshall Islands, Papua New Guinea *, Solomon Islands, Vanuatu, New Caledonia, Fiji, Nauru, Tuvalu, Kiribati, Tonga, Samoa, American Samoa *, Wallis and Futuna, French Polynesia, Niue, Cook Islands, Tokelau and Pitcairn Island *. * not included in our analysis) are experiencing the triple burden of malnutrition—the coexistence of under-nutrition, over-nutrition and micronutrient deficiencies [9,10,11]. Eight of the world’s ten most obese nations are PICTs and the prevalence of diet-related non-communicable diseases (NCDs) is particularly high [12]. NCDs are responsible for around 70% of all deaths in PICTs and have resulted in falling life expectancy in some countries [13]. 

In most Melanesian and Polynesian countries, rates of urban population growth are much higher than in rural areas and some countries now have a large percentage of their population living in urban areas [14]. As a result of urbanization and the growth of food imports, dietary patterns have shifted from traditional diets comprising a variety of fresh fish, tubers and local vegetables toward diets high in fat and sugar [12,15]. These dietary changes have been influenced by multiple drivers including limited access to land within urban centres, low household incomes and the availability of cheaper and convenient, imported and processed foods such as noodles, white rice, and canned fish and meats [12,16]. This ongoing dietary transition, combined with the resulting over-reliance on commercially sourced foods and consumption of a poor variety of foods, has led to the subsequent rise in food and nutrition insecurity and non-communicable diseases [15,16,17]. However, dietary transitions and their impacts unfold differently at local scales and are not adequately captured in national data.

Although aquatic foods play an important role in the diets of Pacific Island people, there are many gaps in the evidence needed to understand the broader contribution to nutritional adequacy and health status in PICT populations [18]. Previous studies have reported consumption of “fish”, but do not provide further detail on species, or on other types of aquatic food consumed. For example, “fish” consumption has been recorded, noting if it was fresh or canned, in Vanuatu [19,20,21] and Solomon Islands [22,23,24]. In one study investigating the percentage of women and young children consuming fresh fish, canned fish, and other seafood, it was noted that a small percentage of women (4%) reported consuming other seafood, principally shellfish [25].

Reporting consumption under the broad category of “fish” belies the great diversity of fish and invertebrates, especially coral reef species, that support coastal fisheries in the region [26]. More than 300 species of finfish are harvested by small-scale fisheries as well as a range of invertebrate species [27], although relatively few species dominate small-scale, commercial reef fisheries in the western and central areas of the region. While invertebrates such as trochus and sea cucumber are commonly harvested for export, many invertebrates including molluscs, crustaceans and cephalopods may be as critically important in the Pacific as in other subsistence fisheries and local markets [28], but there are limited catch statistics available [29,30]. The volume of production from coastal subsistence fisheries is generally greater than that from coastal commercial fishing, and involves hundreds of different taxa, including fish, molluscs, crustaceans, algae and other groups [31]. The potential for aquaculture to contribute to food and nutrition security in the Pacific has been recognised, in particular for pond aquaculture enterprises in peri-urban areas [32]. However, much of the investment made in establishing animal aquaculture has targeted export commodity markets with limited scope for food security [33]. Investments have also been made in diversifying seaweed industries in Pacific island countries with the aim of improving marine-based livelihoods through food and health applications [34]. The variation in aquatic food types, their nutrient composition and patterns of consumption is important from a health and nutrition perspective and represents a significant data gap. 

Although subsistence fishing is important for coastal rural populations, the amount of aquatic food being traded for cash is growing as rural economies become increasingly monetized. The result has been a gradual shift away from fishing for home consumption, or to meet social obligations, towards fishing to generate income [31]. Some of this income is used to purchase other food, including imported processed foods such as canned fish. 

While consumption of aquatic food, both fresh and canned, remains high, public health in the Pacific is not improving. Increasing aquatic food consumption is promoted in many countries for public health benefits, particularly where current consumption rates are low [35]. However, broad promotion of aquatic food consumption on its own is unlikely to result in improved health outcomes in the Pacific Island context. Filling data gaps on the contribution of aquatic food to Pacific diets, based on the availability of different aquatic food types and their nutrient composition, is essential for addressing food and nutrition security. In addition, developing a better understanding of the variation in consumption among countries, across urban and rural divides, and within intra-household distribution of food is critical to improving public health interventions. In particular, understanding consumption of aquatic foods in the context of broader dietary diversity in nutritionally vulnerable groups, particularly women and children, will be key to leveraging aquatic foods to address intergenerational malnutrition [36]. This study contributes to the development of a more holistic understanding of why NCDs and malnutrition remain prevalent amidst such high rates of national aquatic food consumption. 

Recent work has shown there is considerable potential for marine fisheries to help address deficiencies globally through the supply of essential micronutrients [37]. If Pacific countries are to harness the potential of aquatic food to help address the triple burden of malnutrition, developing a better understanding of consumption patterns, and the influence of access to a diverse range of healthy food through local food systems, are critical next steps. The aim of this paper is to examine the variation in consumption patterns and nutrient composition of aquatic foods in the Pacific, to help improve understanding of their contribution to food and nutrition security. For this examination we analysed nutrient composition data and trade data from two novel region-specific databases, as well as consumption data from national and village level surveys for two Melanesian case studies, Vanuatu and Solomon Islands.

## 2. Materials and Methods 

A mixed method approach was used to incorporate data from different sources, including: nutrient composition data from the newly created Pacific Nutrient Database; national level data on aquatic food consumption from Household Income Expenditure Surveys; village-level data on women’s food consumption collected as part of an ongoing diet diversity study in Solomon Islands and Vanuatu; and trade data from the newly developed Pacific Food Trade Database. These diverse datasets are analysed jointly here and methods for each analysis are described below.

### 2.1. The Pacific Island Region and Case Study Countries

The Pacific Island region consists of fourteen independent countries and eight territories located in the western and central Pacific Ocean, made up of thousands of high islands, low islands and atolls [3]. Depending on the type of island, Pacific populations have access to varied local environments that support different types of domestic food production; their geography influences productivity for example [38]. The region experienced a rapid 30% drop in domestic crop production during the 1980s which has not been recovered–these declining trends in domestic food production have happened concurrently with the doubling of food net-imports [39]. The variation in local environments, combined with factors such as distance to markets, can influence diets and nutrient intake. Most of the population is rural but movement from outer islands and rural areas to urban centres is increasing. Many urban centres are coastal with an estimated 95% people living within 5 km of the coast [40].

**Figure 1:**
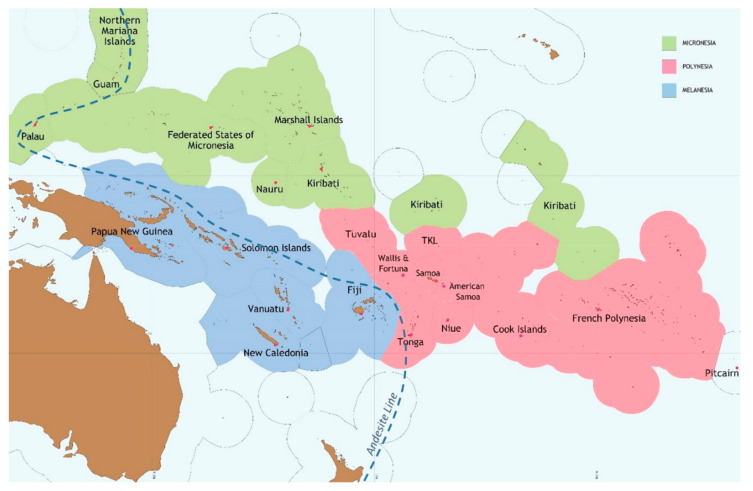
Map of Pacific Islands’ countries and territories.

The diversity in fishing practices, marine resource consumption and dependency on the sea across PICTs is rooted in the various social and physical factors shaping island lives. Early human migration into the region by different ethnic groups from South East Asia and Australasia has formed three distinct ethnographic regions; including Melanesia spanning the western islands to the north and northeast of Australia, and Micronesia and Polynesia spanning the islands respectively north of New Guinea and east into the central Pacific Ocean. In geophysical terms, the Andesite line forms a geological divide that traces a deep ocean trench along the western tectonic shelf and separates the continental islands to its west from the oceanic islands to its east (Figure 1). As such, Melanesia’s large and high-elevation continental islands provide better agriculture conditions, and thus over time have come to support larger, culturally and linguistically diverse populations that maintain a more landward livelihood orientation. The land area and population of Melanesia account for about 98% and 90% of the regions’ total [41], harbouring around 1500 indigenous languages [42]. The smaller more dispersed oceanic islands of Micronesia and Polynesia on the other hand, include low-lying coral atolls with poor soils and volcanic islands that have extreme topography. These islands over time have come to support smaller populations that have settled along coastlines, and exhibit a stronger seaward orientation in their culture and livelihood. Living in a maritime region where, if excluding Papua New Guinea, less than 0.5% (about 88,600 km^2^ of the estimated total 27 million km^2^) of the combined area of the 22 PICTs’ jurisdictions is land [43], rural Pacific islander life reflects an existence characterized by limited social and economic connectivity due to relative isolation of its many islands. (Due to its size, Papua New Guinea skews the region’s population and land area statistics, hiding the region’s predominant island environment. When including Papua New Guinea, about 1.8% of total jurisdiction is land (about 550,000 km^2^ of the approximate 30 million km^2^ total area). Papua New Guinea and New Zealand are not included in this analysis.)

### 2.2. Vanuatu

Three quarters of Vanuatu’s 292,675 population [44] live in rural areas, across an archipelago of 80 islands. Vanuatu is ranked amongst the most natural disaster prone countries in the world, primarily due to frequency of seasonal cyclone events and volcanic activity [45]. Most land is forested, or of extreme topography, and only 12% is estimated to be cultivated. The agriculture sector, including fishing, makes up 26% of the gross domestic product (US$ 802 million) [46]. Major urban populations center around Luganville, on the island of Espiritu Santo, and the country’s capital Port Vila on the island of Efate. Growth of these urban populations is significant, with an estimated 2.9% annual growth [47], putting increased pressure on domestic food production systems to meet demand for food. In rural areas, households derive most of their income and consumption needs from agriculture-based activities, with 32% from sales of agriculture and home-made products at local markets and 39% from subsistence production [48]. On average, over half (56%) of household annual expenditure is on food (62% and 42% respectively for rural and urban populations) [48]. In 2013, the government of Vanuatu set out a policy direction to address food and nutrition security challenges in the country. Therein it “outlines a holistic approach to address all key elements of food security along the food chain from farm to fork, i.e., from primary production, processing, trading, marketing, preparation and consumption”, with emphasis put on “investing in improving and increasing production and productivity of the agricultural and fisheries sectors” [49] (p. 12).

### 2.3. Solomon Islands

Solomon Islands is an archipelago of more than 1000 islands in the south-west Pacific Ocean with a population of 652 856 [44]. The country experienced a period of civil conflict that was brought to an end through international intervention in 2003. Since then the country’s economy has grown at an average annual rate of 5.5 percent [50]. However, this metric masks a development struggle. The country is ranked 152nd on the human development index and is among the least developed countries in the world [51]; approximately 13% of the population is classified as poor [52] and this is projected to rise anywhere from 2–12 percent due to the most recent external shock—COVID-19 [53].

Most economic activities in the country takes place in the informal sector, which includes small-scale agriculture and fisheries [54]. Most Solomon Islanders live in rural, coastal communities where fish is the primary animal-source food and gardens provide root crops and vegetables for household needs and income generation [12].Growing conditions are favorable for a range of crops, and the majority of the population is engaged in agriculture in some way on the 1.1 million ha of agricultural land in use [55]. Fishing is widespread and productive, with 68% of households in rural areas reporting catching fish or shellfish, and one-third of all households in urban areas engaged in fishing activity [56]. Despite this largely subsistence mode of living, rapid population growth, shortages of arable land in urban environs, declining fish stocks and cheap, low-quality food imports create challenges for nutrition security [16].

### 2.4. Nutrient Composition

The Pacific Nutrient Database (PNDB) [57] is a new database developed to address the need for a standardised method for linking data between the Household Income Expenditure Surveys (HIES) and nutrient composition data for foods consumed in the region. The PNDB matched 822 food items from the HIES with their respective macro- and micro-nutrient composition available from the literature. The PNDB presents values per 100 g edible portions for total energy, all macronutrients, fibre, and 17 micronutrients. The PNDB authors’ process was limited by the availability of secondary data, where items were matched to their closest taxa or description; not all micronutrients are represented in the PNDB for this reason. For example, while aquatic food is a rich source of marine long chain omega -3 fatty acids, iodine and selenium [58,59], these nutrients are absent from the current iteration of the PNDB. Other key nutrients relevant to aquatic food as a source are included, such as protein, calcium, iron, zinc, vitamin A and vitamin B12. As such, nine nutrients are displayed within this paper based on their inclusion in the PNDB, their relevance to aquatic food and the purpose of the study. 

At present there are 42 individual aquatic food items, or species, included in the PNDB. These individual species were aggregated into taxonomic groupings and the mean value was presented to demonstrate the nutritional composition of different aquatic food groups. The range between highest and lowest value was also determined for each aggregate group containing three or more species. The range was included, rather than standard deviation, as the number of individual species within aggregated groups were too small to apply standard deviations. Further information on grouping of species is available in the supplementary material (Table S1). 

### 2.5. Consumption Data

Consumption of aquatic foods was explored at different scales using two data sources. National level data on consumption of different aquatic food groups was quantified using HIES data (Section 2.5.1), and village-level data on the number of food groups consumed by women was sourced through dietary diversity surveys (Section 2.5.2). 

#### 2.5.1. National Consumption of Aquatic Foods

Mean annual per capita apparent consumption of a range of aquatic foods was calculated from HIES data for Vanuatu and Solomon Islands, at a national level, as well as by urban and rural settings. The 2012–13 Solomon Islands HIES was implemented over the course of 12 months from October 2012 and was stratified by urban and rural areas in all 10 provinces, except for the province of Rennell and Bellona, which has only rural clusters, and Honiara, which has only urban clusters. The 2010 Vanuatu HIES was implemented in all provinces over the course of four months from October 2009 (See also http://www.statistics.gov.sb/statistics/demographic-statistics/household-income-and-expenditure-surveys, https://microdata.pacificdata.org/index.php/catalog/731 and https://microdata.pacificdata.org/index.php/catalog/727/ for further detail on design and implementation of the surveys.).

As with other HIES analyses at the national level, we used income and expenditure on specific food groups as proxies for acquisition and consumption. Apparent consumption data (hereafter more simply consumption) from the HIES were converted to per capita edible portions for analysis using the conversion factors from the PNDB. We recognize the limitations of using HIES to generate proxies of per capita consumption but in the data sparse landscape of the Pacific region they provide an important and under-utilized source of evidence [60,61].

#### 2.5.2. Village-Level Food Consumption

Data on food consumption, including aquatic food, was collected as part of an ongoing diet diversity study in Solomon Islands and Vanuatu [25]. The study applied the internationally validated tool, the Minimum Dietary Diversity for Women of Reproductive Age (MDD-W) [62], as an indicator of the micronutrient adequacy of the diets of women of reproductive age (15–49 years). The MDD-W is a categorical indicator of the proportion of women who consumed at least five out of ten food groups within the past 24 h recall period. The tool does not quantify amounts of the reported food group consumed or quantify intakes of processed/excess non-nutritious foods. Instead, it is a measure of the number of different food groups consumed, as recalled by respondents in the 24 h recall period. The MDD-W tool and study method detailed by Albert et al. [25] was further applied in an extended study in seven rural sites in 2018 and 2019. This previously unpublished data on diet diversity scores based on food group recall of the second study are presented within this paper. 

The 24 h recall method for MDD-W was carried out with women aged 15–49. The survey team convened a community meeting to introduce the survey and its objectives to understand dietary patterns in the village. Eligible and consenting respondents were then randomly recruited for the survey, representing at least 50% of households in each village surveyed (*n* = 260). The total sample across sites was 260 women of reproductive age. Data were collected at different times of the year (Site A and B in September and November respectively, Sites C and D in March, Sites E, F and G were sampled on two occasions in February/March and in April). Seasonality is a major feature in rural growing practices and food production. The north-western trade winds, *koburu*, extends from January to March, which is generally the lean period in many of the sampled sites [63]. Our sampling of consumption patterns is therefore likely influenced by season.

Within this study, we have modified the presentation of the ten MDD-W food groups [62] by separating aquatic foods from the meat, poultry, fish group. However, the total diversity score out of ten is calculated per the original MDD-W method as a combined fish and meat group.

### 2.6. Trade of Aquatic Foods

PICT import and export of aquatic foods is limited, in terms of quantity, primarily due to the significant domestic harvest. However, two aquatic food groups do comprise substantial trade quantities; tuna and canned fish. The dominant fishery in the Pacific, in terms of total catch volume is the oceanic tuna fishery. A large portion of the fishery occurs within PICT national waters, with harvesting conducted predominantly by foreign-flagged vessels and transhipped to countries outside the region. Only a small portion is harvested by domestic vessels. These fisheries provide significant benefit to PICTs, including foreign fleet revenues and contributions of landed catch to GDP [64]. We present existing tuna harvest data (Pacific Islands Forum Fisheries Agency (https://www.ffa.int/catch_value), in terms of potentially available grams per capita per day to consider the potential role of commercial tuna in Pacific diets. Papua New Guinea was excluded from analysis because it is more agriculture focused and has a human population size which exceeds all other PICTs combined, and would, therefore, skew interpretation. Canned fish, including tuna, mackerel, sardines and pilchards comprise an increasing portion of Pacific diets [65]. Here we assess apparent consumption of canned fish import data using the new Pacific Food Trade Database (PFTD) [66]. The PFTD was developed to better understand international trade in food and beverages in the region. The database is based on data from BACI international trade database, which amends Comtrade data [67], including the use of mirror data to fill gaps. Development of the PFTD included significant expert input to identify categorical errors (e.g., commodity, exporter, and importer) and imputation to address quantity errors. The resulting database, covering all food and beverage trade for 18 PICTs for the years 1995–2018, addresses substantial error in global data and delivers the benchmark in reliable food and beverage trade data for the region. 

## 3. Results

### 3.1. Nutrient Composition of Aquatic Foods in the Pacific

The available nutrient composition data in Table 1 represents only a small proportion of the thousands of aquatic foods available in the Pacific, yet it demonstrates that large variation exists in the nutrient composition of aquatic foods commonly consumed in the region. The results also highlight consistency in valuing aquatic foods as a nutrient dense food group with large variation across specific micronutrients. 

Aquatic food is an important source of quality protein; however, it is also rich in a variety of highly bioavailable micronutrients, of which several are of public health concern in the Pacific Island region. Small pelagics, crabs and crayfish, turtle and canned fish, for example, are good sources of calcium, containing 725, 143, 100, 154 mg/100 g respectively. Small fish that can be consumed whole including the bones, such as sardines, and those softened in canned fish are especially rich in calcium. Some aquatic groups examined showed substantial range in nutrient content between species, for example, bivalves, such as oysters contain high amounts of calcium, while others such as sici-shells contain very little (see Table S1). 

Bivalves and gastropods, seaweed and small pelagics are important sources of iron. Small pelagics, bivalves and gastropods contain similar amounts of iron (4 mg/100 g), which is almost seven times higher than the amount contained in demersal and reef fish. Seaweed contains 8 mg/100 g which is double the amount contained by pelagics, bivalves and gastropods. Cephalopods, bivalves and gastropods, and small pelagics are very rich in vitamin B12 (10.2, 9.4, 8.3 µg/100 g respectively), in comparison to other groups, for example, tuna (1.1 µg/100 g) and prawns (1.5 µg/100 g). Small pelagics (consumed whole) also provide more vitamin A (106 µg RAE/100 g) than all other aquatic groups examined (Table 1). All groups examined, except sea urchin and seaweed, contained a meaningful contribution to daily requirements per 100 g portions important for cognitive function and development. 

Large nutrient variations are seen within the different types of canned fish. This is due to the varied species types and species-specific nutrients, as well as variance in ingredients such as oil, brine, salt, or tomato and the recipe of the final product. The higher mean value for fat in canned fish is from the oil added to some canned varieties. 

Aquatic food can also contain high levels of sodium, particularly processed and canned fish. The high sodium content, and variation of recipes of processed products, must be taken into consideration in recommendations to increase aquatic food intake from processed forms. 

### 3.2. National Consumption of Aqautic Foods

Apparent consumption of aquatic foods is high in PICTs, however, consumption trends are not homogenous and the volume and type of aquatic food consumed varies among sub-regions, countries and within countries. For example, variation exists between rural and urban consumers (Table 2) and between communities, partially as a result of availability. Solomon Islanders each consume, on average, 73 kg of aquatic food (all types) per year. In contrast, 14 kg of aquatic food is consumed per person per year in Vanuatu, which is less than the global average of 20 kg/p.c./year [68]. Within Solomon Islands, rural consumption of pelagic fish alone is equivalent to the global average consumption of aquatic food, with an additional 63 kg/p.c./year of other fish and seafood consumed. Urban consumption of 48 kg/year of aquatic food is still high in terms of the global average, but is much lower than rural consumption of 83 kg/yr. Different types of aquatic food make different contributions to consumption, for example, shellfish account for over 15% of total consumption in Solomon Islands and 4% in Vanuatu. Within Solomon Islands, shellfish account for 17% or rural aquatic food consumption and 7% of urban consumption (Table 2). Canned fish is consumed more by urban than rural consumers in both Vanuatu and Solomon Islands. Urban and rural consumers typically have different access to healthy and nutritious food, as well as differing dietary gaps. As demonstrated by this variance in volumes and types of aquatic foods consumed, further food and nutrition requirements must be considered within a localized context to inform relevant and appropriate interventions to address dietary gaps. 

### 3.3. Individual Consumption

The majority of Pacific Islanders live in rural settings where food access and availability vary according to factors such as the amount of arable land, distance from nearest market, population size and coastal habitat. The result is substantial variation at village and household level in the diversity of foods consumed, as well as the species of aquatic food types consumed. 

**Figure 2:**
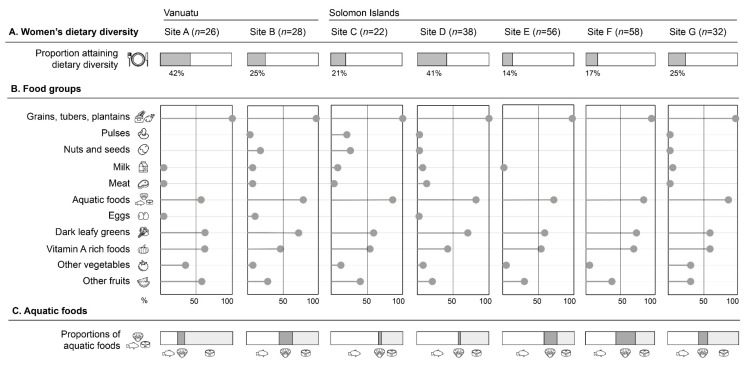
Village-level consumption based on 24-h recall of women of reproductive age (*n* = 260) [25]. (**A)**. Proportion of women attaining minimum dietary diversity (five or more food groups); (**B**)**.** Frequency of food group consumption in 24-h recall; (**C**). Proportion of three categories of aquatic foods (finfish, invertebrates, canned tuna) reported in 24-h recall. Foods are grouped based on their nutritional content as defined in the MDD-W index method [62]. The group ‘Vitamin A rich foods’ includes other Vitamin A rich fruit and vegetables not included in other groups. ‘Aquatic foods’ includes animal sourced aquatic foods only. Together, meat and aquatic foods (separated for this figure) contribute as an aggregate score out of one in calculating total diet diversity scores.

Women’s dietary diversity was low with 14–42% meeting the minimum dietary diversity score of five across the seven rural sites in Melanesia included in our study (Figure 2A). Some similarities between women’s diets were evident across sites. Grains, tubers and plantains were consumed by virtually all respondents (96%) (Figure 2B). Products in this group include food such as locally grown tubers as well as imported rice. Dark leafy greens, Vitamin A rich foods, and other fruits were consumed by over 50% of respondents at all sites. Consumption of other vegetables was generally low. The three major food groups with low consumption included pulses, nuts seeds, milk/dairy and eggs. 

Of the respondents that reported consuming meat, poultry, and fish, the majority (78%) of this consumption was from aquatic foods. While finfish, including reef, mangrove and pelagic species, were important for consumption across all sites (Figure 2C), the type of aquatic food consumed varied between sites. For example, canned tuna was the main source of aquatic food at site A in Vanuatu, where there are few lagoons to harvest aquatic food from and limited arable land. However, fishers have access to deep oceanic high-value fisheries and fishing activity in this site is predominantly commercially-oriented, where aquatic food is caught to generate income rather than for local consumption. 

In contrast, invertebrates, including shellfish and crabs, were an important source of aquatic food at sites B, E, F. These sites are complex habitats where fishers have access to shallow reef, lagoon and/or mangrove habitats to harvest food for local consumption. They are also located far from provincial market centres and so exhibit a dietary composition largely reflecting the growing and fishing conditions at their location, with addition of long shelf-life foods bought at rural stores (e.g., rice, biscuits and canned tuna). 

### 3.4. Movement of Nutrients from Aquatic Foods through Imports and Exports

#### 3.4.1. Canned Fish

**Figure 3:**
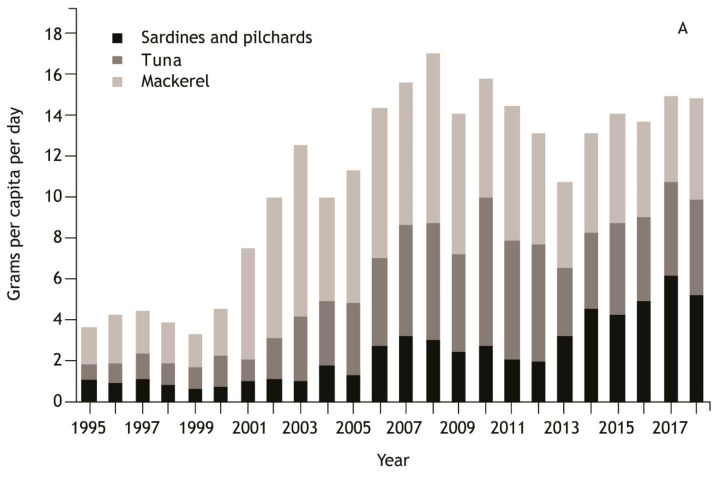

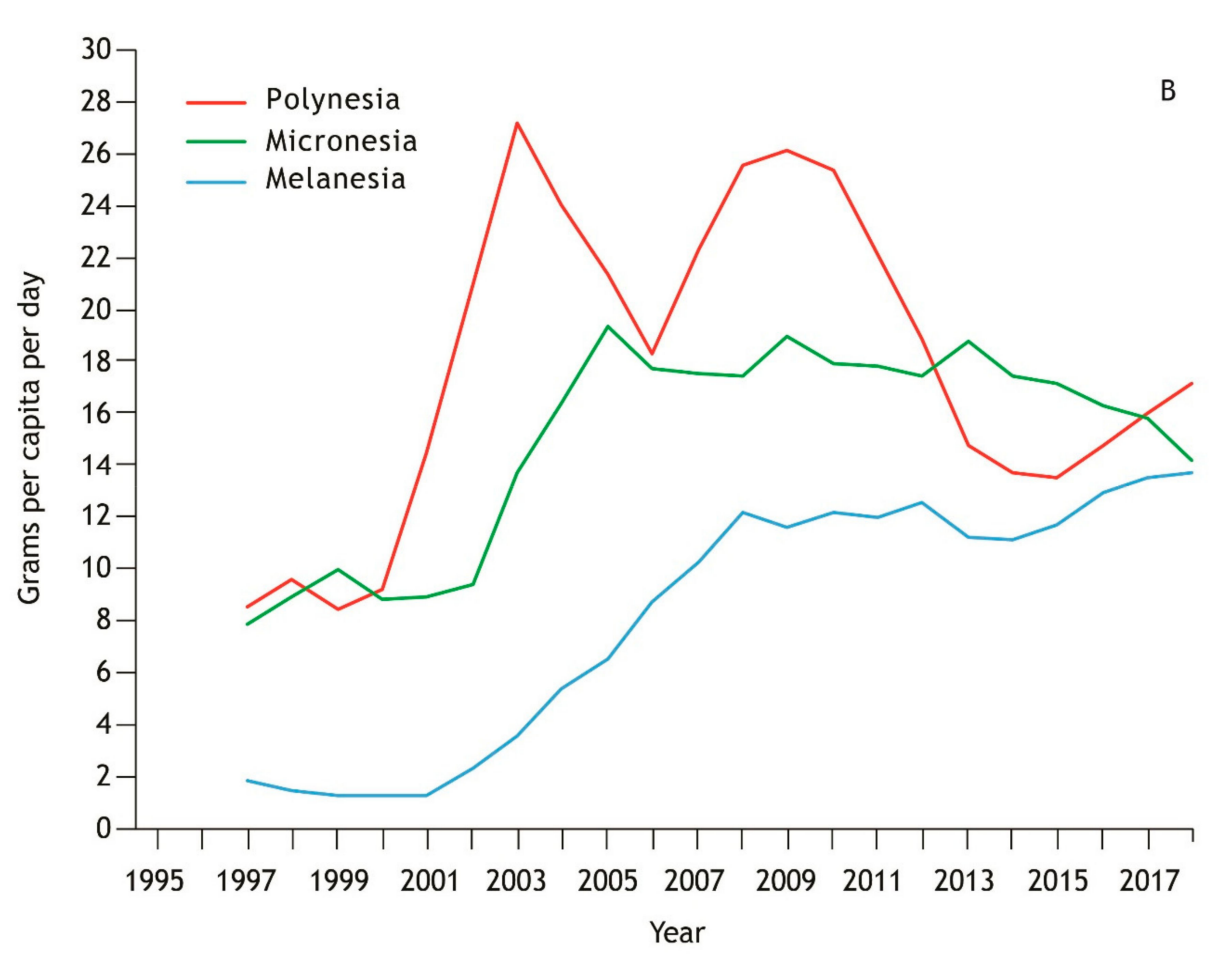
Per capita import (apparent consumption) of canned fish to 17 Pacific Island Countries and Territories (full list available in supplementary information, Papua New Guinea excluded because of its relative dominance in the data set) ncluding (**A**) regional trend by fish types and (**B**) total import by sub-region. Estimates exclude other minor quantities of canned fish including salmon and herring. Sub-regional trends are a 3-year moving average and include all canned fish types included in the regional estimate. Edible portions are likely to be 0.7 to 0.85 of the estimates shown due to inedible portions (e.g., brine, oil and cans) and variation in proportion of fish by total weight across different fish types and processing methods (see Table 1). Canned fish data from the Pacific Food Trade Database [66]. Human population data from UN Population division (https://www.un.org/development/desa/pd/). .

The majority of aquatic food consumed in PICTs is caught domestically, however, aquatic food imports play an increasingly prominent role in the diets of Pacific Island people [65] (Figure 3). Canned mackerel, tuna, pilchards and sardines comprise the bulk of consumed canned fish. These fish species have different nutrient compositions, and further variance from ingredients within the final canned product (Table 1). 

There has been a significant increase in imports of canned fish to the region over the last two decades, with a rapid increase from 1999 to 2007 and a subsequent plateau, mostly imported from China and Thailand [66]. Total regional consumption of canned fish increased from 3.6 to 14.8 g per capita per day, between 1995 and 2018 (Figure 3). Regional import of canned mackerel (grams per capita per day) has increased from 1.8 in 1995 to 4.9 in 2018, with a peak of 8.3 in 2008. Import of canned tuna (grams per capita per day) increased nearly 6-fold, from 0.8 in 1995 to 4.7 kg in 2018. In consumption terms, this is an underestimate as it excludes tuna canned in Fiji and Solomon Islands, and sold domestically, which contributes substantially to total consumption [65]. Across the region, canned sardine and pilchard imports increased from 1 in 1995 to 5.2 g/capita/day in 2018. Importantly, there are significant differences in per capita imports between sub-regions (Melanesia, Micronesia, and Polynesia) (Figure 3). 

#### 3.4.2. Oceanic Tuna Fishery

**Figure 4:**
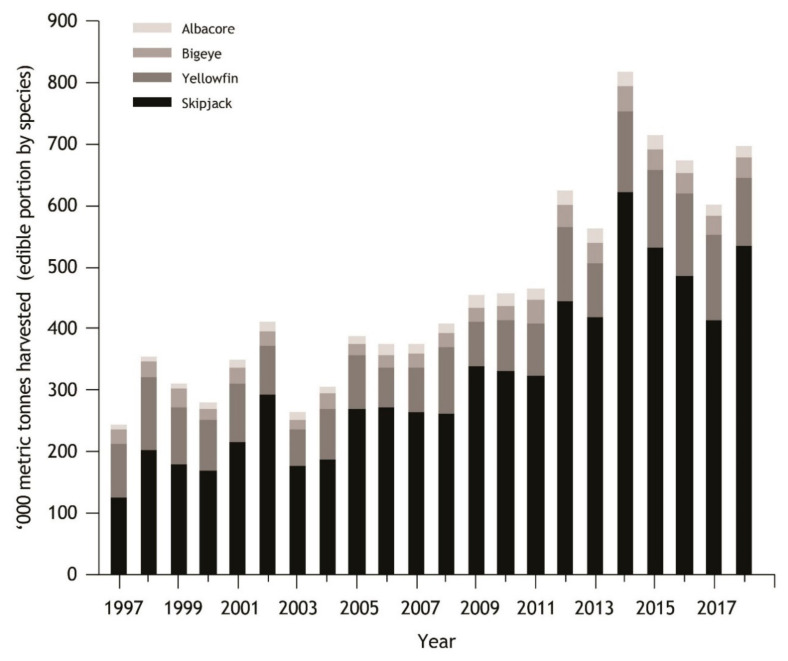
Temporal trend in total edible portion of registered catch (1997–2018) within PICT (same PICTs as Figure 3) national waters. Total catch was calculated as the total catch of Albacore, Bigeye, Skipjack and Yellowfin tuna by domestic and foreign-flagged vessels multiplied by 0.58 [53] to derive a measure of edible harvest. Tuna data from Pacific Islands Forum Fisheries Agency (https://www.ffa.int/catch_value). Human population data from UN Population division (https://www.un.org/development/desa/pd/).

Between 1997 and 2018 over 17.5 million metric tonnes of the four dominant species of tuna (Albacore, Bigeye, Skipjack and Yellowfin) were harvested from national waters of the 17 PICTs included here, with Skipjack accounting for the majority of the harvest (Figure 4). There are, however, drastic differences in harvest, on a per capita basis, between Pacific sub-regions. In 2018, total catch in grams per capita per day, was 80, 440, and 4630 in Melanesia, Polynesia, and Micronesia respectively. While there are large differences in per capita harvest rates among sub-regions and individual PICTs, in edible portion terms (0.58 * total weight) [53], the catch harvested has risen from around 250,000 metric tonnes in 1997 to 700,000 metric tonnes in 2018. These quantities relative to PICT populations and economies represent a profound asset in terms of revenue, although in terms of food and nutrition security, the majority of this nutritional value is exported from the region. The estimated need for tuna for good nutrition across the region by 2035 is estimated to be 87,500 tonnes [69].

## 4. Discussion

Aquatic foods play an essential role in food and nutrition security in the Pacific region, however, they are not a homogenous food group in terms of nutrient composition. The variation in nutrient composition indicates that some groups may be more suited to addressing specific health issues in the Pacific region than others. Consumption of aquatic foods is highly variable among sub-regions, countries and within countries, with people consuming what they can access. The majority of aquatic food consumed in PICTs is caught domestically, however, aquatic food imports play an increasingly prominent role in the diets of Pacific Island people. Fisheries, such as the oceanic tuna fishery, represent a profound asset in terms of revenue, although in terms of food and nutrition security, the majority of this nutritional value is exported from the region. While most Pacific Islanders consume large amounts of aquatic food, this consumption is part of a diet that does not meet the recommended dietary diversity. More data is needed on the micronutrient content of locally relevant aquatic foods, as well as on whole diet consumption at regional, national, household and individual levels, to improve public health, particularly in regions where aquatic food consumption is already high, yet overall dietary diversity remains low. 

### 4.1. Aquatic Food Is Not a Homogenous Group

Unlike the term “chicken” or “pig”, which denotes an animal from a single genus (*Gallus* and *Sus* respectively) [70], “aquatic food” and “seafood” include multiple taxa of biologically divergent animals, and often plants, from freshwater and marine habitats. As a result, the nutrient composition of different aquatic foods varies considerably and the nutritional value of aquatic food far exceeds its common description as a quality protein source. Aquatic food is an important source of micronutrients, of which several are of public health concern in the Pacific Island region. Sardine, crabs, crayfish, turtle and canned fish, for example, are good sources of calcium, highlighting these alternate calcium sources of importance as compared to the negligible dairy intake reported by the diet diversity respondents in this, and other, research [25]. Iron rich species such as bivalves and gastropods, seaweed and sardines, are important sources of iron which can contribute to meeting iron requirements in Pacific populations suffering diet and parasitic related anemia [7]. Aquatic foods have also been identified for provision of high biological vitamin A, as well as calcium, iron and zinc and as a target food for combating these micronutrient deficiencies in S/SE Asia [59,71]. 

Our results, based on what is currently available in the PNDB, support current evidence that there is considerable variation in the nutrient composition of different aquatic food groups [37,72]. This variation is of importance in nutrition sensitive food systems and food-based dietary guidelines, whereby specific aquatic food sources can be targeted to fill particular nutrient gaps. However, the ability to identify aquatic foods to target nutrient gaps is limited by the lack of data on the concentration of nutrients and contaminants in aquatic foods, especially in low- and middle-income countries [73]. Greater understanding of micronutrient content of locally relevant aquatic foods is critical to formulating advice around consumption and harness the potential of aquatic foods in addressing the triple burden of malnutrition in the Pacific, and elsewhere. 

### 4.2. Integrating Nutrient Compostion Information with Consumption Patterns

Combined with the need for greater understanding of nutrient composition, is the need for information on quantified consumption at regional, national, household and individual levels, and the factors that drive differences in consumption patterns. In particular, better understanding of the needs, access and modes of acquisition of rural and urban consumers and vulnerable groups is required. Local environments and cultural contexts have a major influence over the types of aquatic foods that can be accessed and consumed. For example, our results identified that in areas where there are few lagoons to harvest aquatic food from and limited arable land, canned tuna is an important source of aquatic food. In contrast, invertebrates, including shellfish and crabs, were an important source in areas located far from provincial market centres, where fishers can harvest aquatic food from shallow reef, lagoon and/or mangrove habitats. 

Aquatic food consumption is strongly influenced by local environments, as well as local customs, which can determine whether the aquatic foods are consumed or sold. For example, some diet data analysed as part of this study were collected during a “kustom” or traditional fishing season, which may have influenced results. In addition, for some PICTs, religious dietary proscriptions limit consumption of different types of animal source food (e.g., the Seventh Day Adventist church, a major faith in Solomon Islands, prohibits consumption of shellfish). 

The Pacific Community (SPC) Public Health Division advises that, on average, each person in the region should eat about 35 kg of fish per year. This value reflects aquatic food contributing up to 50% of the daily protein intake recommended by the World Health Organization for good nutrition [74]. National consumption in some PICTs already exceeds of this amount, yet major health problems continue to plague a large proportion of the Pacific population [9,75,76]. While most Pacific Islanders consume large amounts of aquatic food, this consumption is part of a diet that does not meet the recommended dietary diversity. With exceptions, such as Vanuatu and Papua New Guinea, recommendations to increase “fish” consumption at national levels are unlikely to benefit the health of the population. Contemporary national level recommendations around “fish” or “seafood” now need to take in to account the varied needs of different population groups, the food groups that are absent from their diets and the relative nutritional benefits of different aquatic foods. For example, the village site in Solomon Islands with the lowest dietary diversity score showed almost all people ate aquatic foods including fish, shellfish and canned tuna. In this situation, health advice should be to maintain consumption of aquatic foods in conjunction with messages to increase the diversity of other foods consumed. In contrast, where dietary diversity is higher, but consumption of aquatic foods is lower and less diverse, health messages should focus on increasing diversity of aquatic food consumption for improved nutrition rather than increasing consumption per se. Achieving Recommended Dietary Intakes (RDIs) for specific nutrients through one source of aquatic food, for example a species group such as tuna, is unrealistic. RDIs should instead be reached through a combination of different food groups, including a diverse range of aquatic food.

### 4.3. Aquatic Food as Part of the Pacific Food System

Advice to increase consumption of aquatic foods will also need to be considerate of the broader transition in diets occurring in Pacific countries, including consumption of less healthy foods. Progress in shifting dietary and health trajectories toward better outcomes will require nutrition-sensitive interventions aimed at the structural drivers of national food systems and the external food environment [25,77].

Broader development (e.g., infrastructure, communication, banking, public services) is lacking, so the transportation and distribution of highly perishable fresh foods from the garden or sea to the urban market place are difficult and expensive. This is a driving factor to the influx and acceptance of refined foods like rice and flour or long shelf-life convenience foods, all of which paradoxically tend to outcompete domestic grown crops and fish in price, access and availability in urban settings [78]. Enhancing storage, processing, and distribution of food commodities is vital in mitigating food and nutrition security impacts from changing food production patterns and during the current COVID-19 crisis [39].

Aquatic foods also play a key role in disaster recovery in the Pacific, where terrestrial-based food and income generation capacity has been reduced. The capacity for marine resources to support recovery, however, is dependent on factors such as market access, as well as fishing skills and technology in many sectors of the community [79,80,81].

### 4.4. Effects of Aquatic Food Trade on Nutrition

#### 4.4.1. Imports

Most of the aquatic food consumed in the Pacific is caught domestically, however, consumption of imported aquatic food is increasing, particularly canned fish. Paradoxically, although there are major canneries in Solomon Islands, Fiji and Papua New Guinea, much of the tuna caught in the EEZs of PICTs is transshipped to Asia for processing [65]. The contribution of canned fish to national security in the region is mixed, and in need of more research to better place it within national and local dietary patterns. Canning an otherwise highly perishable food greatly increases access to aquatic food for people that do not have reliable access to fresh fish or refrigeration, but canned fish can be high in salt and consuming high amounts can lead to negative health implications [82]. However, unlike other processed foods high in sodium, which can be of overall low nutritional value, aquatic food also contains other nutrients. An opportunity exists to revisit sodium guidelines to ensure that canned fished does not contribute excess sodium.

Identifying differences in per capita aquatic food imports among sub-regions (Melanesia, Micronesia, and Polynesia) and individual PICTs, is important for understanding trade-derived nutrition at the community and urban/rural scale. Reviewing population-level trade in food through a nutrition lens deepens the understanding of potential micronutrient sources. If a population were deficient in calcium intake, for example, canned fish such as mackerel and other small pelagic species with bones, could be promoted. If canned fish is not disaggregated by species and recipe type, the nutritional value could be largely over- or under-estimated. Future studies examining food availability of specific canned fish types from trade data, converted to respective micronutrient composition, can help to ensure appropriate estimation of trade derived nutrition.

#### 4.4.2. Exports

Unlike other aquatic foods, the majority of tuna caught in Pacific waters is currently exported. There has been considerable debate over the food security costs and benefits for developing countries engaging in aquatic food trade [83,84]. Hicks et al. [37] identified the need for more research on the relationship between potential nutrient supply from fisheries and international trade and foreign fishing in countries with populations at risk of dietary deficiency. Nash et al. [85] examined the impact of growing global redistribution of fish products on current and future supply of fishery-derived nutrients. They found that international trade contributes to inequities when considering micronutrient supply and that countries currently benefiting from trade and foreign fishing tend to be more vulnerable to future changes in nutrient supplies. An important part of the aquatic food and nutrition story in many PICTs is understanding the quantity of nutrients leaving the region without being landed, in particular the micronutrients needed to overcome stunting, or other deficiencies. Determining the right balance between efforts to divert aquatic food from export markets for domestic consumption, and income generation from international fleets, is an area requiring further investigation [80]. The cost-benefit of such comparisons will need to be measured in terms of public health outcomes.

### 4.5. Future Opportunities and Constraints for Aquatic Foods and Nutrition

#### 4.5.1. Data Gaps

There remains a paucity of nutrient composition data for the Pacific. While the PNDB makes an important contribution to improving data availability, many of the species included in the PNDB are not Pacific species or local samples. Several key nutrients are absent from the current iteration of the PNDB. Our finding that there are large variations in the composition of different species and aquatic food groups highlights that this is an important gap in the literature. The integration of local catch data (that can be disaggregated by species) is much needed to provide a more accurate picture of catch composition and the macro/micronutrients sourced through the consumption of aquatic food. Further sampling of species from both wild capture fisheries and aquaculture is also needed to strengthen the empirical nutrition composition data for aquatic foods in the Pacific region.

#### 4.5.2. Addressing the Predicted Supply Gap from Coastal Fisheries

Coastal habitats that have traditionally provided most of the aquatic foods caught by small-scale fishers are predicted to be progressively degraded by ocean warming and acidification, leading to declines in catch potential [86,87]. These impacts are predicted to occur alongside existing pressures, such as overfishing, erosion and siltation of coastal ecosystems from logging, and mangrove clearing [88] as well as increased coastal urban development, population growth and coastal pollution [86,89]. For many PICTs, the gap between the aquatic foods required for food and the amount of aquatic foods that can be harvested sustainably from reefs will increase considerably [1,69]. The reduced availability of aquatic food from coastal fisheries may be exacerbated by trade disruptions to imports, as evidenced by the Covid-19 pandemic [39]. Proposed solutions to address this supply gap include increasing consumption of canned fish and landing more tuna and bycatch in PICTs from off-shore fisheries [69].

In terms of volume and calories, based on the data presented here, all Pacific sub-regions could increase the availability of aquatic food based solely by landing, rather than exporting, more of the tuna harvested in national waters by registered vessels. While landing more tuna may help contribute to filling the supply gap from declines in coastal fisheries, it may not have the same nutrient densities as the aquatic foods it replaces. Higher quantities of tuna may need to be consumed to get the same amount of a micronutrient and eating this volume of tuna may be unrealistic. A large volume of tuna is already consumed domestically, from a number of sources including domestically produced and imported canned tuna, products entering the region from foreign-flagged tuna vessels, catch from domestic commercial fleets, and coastal artisanal capture from various methods including the use of fish aggregation devices (FADS). However, the total quantity of tuna consumed across PICTs, or for individual PICTs is not accurately known. Such information is essential for measuring the success of the food security goal of the Regional Roadmap for Sustainable Pacific Fisheries [90], which aims to increase access to tuna for domestic consumption by 40,000 tonnes between 2015 and 2024.

Increasing the availability of tuna for Pacific consumers may be an important option for overcoming undernutrition where access is prioritized for vulnerable groups. Given the large differences in per capita harvest rates between Pacific sub-regions and individual PICTs, an understanding of the geopolitical heterogeneity of harvestable tuna will be essential for understanding the potential role of the fishery in meeting future dietary requirements in the region [64]. Other fisheries including inshore finfish and invertebrates, which already contribute to local livelihoods and food security, can potentially play a greater role in food and nutrition security in the Pacific. Inshore species have a varied nutrient composition and together with tuna present a nutrition sensitive approach to increase the supply of aquatic foods. In addition, invertebrates are predicted to be impacted by climate change to a lesser degree than other aquatic foods [26], and future dietary advice will need to consider these eventualities. Where consumption of these species is currently low, efforts to promote consumption may be needed, particularly to target nutrient deficiency in vulnerable groups. Developing messaging around “culturally significant local aquatic foods” may help promote consumption via targeting foods significant to a local environment that may not currently have a place on the “daily plate” for various reasons. Linking these foods with their high nutritional value could potentially increase their consumption. For example, the small mud whelk found in mangroves is often considered a “poor persons food” i.e., people collect it when they have nothing else. If this food source was celebrated for its nutritional value, it may help increase its consumption. Care would also need to be taken to ensure overexploitation did not occur.

Aquatic foods may be substituted with other animal source foods if supply of aquatic foods is reduced. Understanding consumption patterns and the nutrient composition of aquatic foods will provide an evidence base for national context food group substitutions, such as aquatic food portion within respective animal source foods portions and providing alternate non-diary calcium sources. These data will also inform culturally appropriate food-based dietary guidelines (FBDGs). Customizing FBDGs to local food practices, preferences, availability and access, with consideration of the local food system and health education, will be necessary to best support healthy diets.

#### 4.5.3. Addressing Contaminants

As well as being an important source of micronutrients, aquatic food is a source of contaminates, including metals, persistent organic pollutants (POPs), and plastics accumulated from the marine environment [73]. Developing a better picture of variability in aquatic foods is also important for understanding the effects of these contaminants, as well as toxins which cause poisoning such as ciguatera. Mercury levels are typically low in fast growing species but are much higher in larger fish, such as some tunas [91]. Excess consumption of fish with high mercury levels, particularly by pregnant women and children, is harmful [92]. Understanding mercury contamination and safe levels of consumption for each species and size of tuna in PICTs will become increasingly important from a public health perspective if more tuna is landed nationally, rather than exported [93].

Understanding the temporal and spatial variation in Ciguatera Fish Poisoning (CFP) cases will be important in predicting the future role of coral reef fish in local diets. CFP, the most frequently reported seafood toxin illness in the world, can produce a diverse array of complex and debilitating symptoms [94]. CFP is caused by consumption of coral reef associated fish contaminated by ciguatoxin and related toxins from dinoflagellates (microalgae) and cyanobacteria. The toxin bioaccumulates up the food web, with particular fish species being more prone to having higher levels of ciguatoxin [94]. Presence of ciguatoxic fish not only causes ill-health but can alter fishing practices and overall fishing effort which reduces local fish consumption. Public health challenges, in terms of both CFP symptoms and nutrition have worsened over the past 35 years, and are expected to continue to do so as the coastal habitats continue to degrade [95]. In addition to CFP, scrombroid syndrome can result from eating “spoiled fish”, particularly tuna and other, mostly pelagic species, in the family Scombridae. While the syndrome has been reported in local hospitals in Solomon Islands, it is generally considered an underreported form of food poisoning [90]. Improving fish hygiene and ensuring food safety and quality along supply chains will be essential components of improving the contribution of aquatic foods to nutrition.

## 5. Conclusions

General messages to increase consumption of “aquatic food” will not be adequate to improve nutrition and health in the Pacific region. For aquatic foods to best contribute to food and nutrition security in the Pacific region, and more broadly, consumption advice needs to be shaped to match the type of aquatic food already being consumed, its nutritional composition, and its place in contributing to dietary diversity with other foods, both domestically produced and imported.

## Figures and Tables

**Table 1:** Nutritional composition of aggregated aquatic food groups consumed in the Pacific (Nutrient values are based on 100 g edible portions; nutrient composition data adapted from the Pacific Nutrient Database).*

Aquatic Food Group	Energy (kcal)	Protein (g)	Fat (g)	Sodium (mg)	Calcium (mg)	Iron (mg)	Zinc (mg)	Vitamin A (µg RAE)	Vitamin B12 (µg)
Tuna (*n*= 4)	164 (77)	25 (3)	5.4 (8)	38.3 (14)	5.5 (6)	1.2 (0.9)	0.5 (0.23)	54.8 (58)	1.1 (1.4)
Small pelagic fish (*n* = 1)	105	19.7	2.9	665	725	4	3.1	106	8.3
Large pelagic fish (*n* = 2)	132	21	5.2	110	14	0.8	0.7	31	0.9
Demersal/reef fish (*n* = 3)	101 (28)	19.8 (0.3)	2.2 (3.1)	78.3 (11)	25.3 (21)	0.6 (0.3)	0.6 (0.1)	30.3 (2)	1.7 (1.6)
Elasmobranchs (*n* = 2)	97.5	22.6	0.8	97.5	11.0	1.0	0.5	12.0	1.1
Prawn/shrimp (*n* = 2)	92	20.4	1.2	249	89	1.6	1.31	27	1.5
Crabs/crayfish (*n* = 5)	76 (18)	15.7 (5.2)	1.4 (0.8)	279 (244)	143 (139)	1.3 (1.1)	3.5 (0.6)	16.2 (21)	2.9 (3.9)
Bivalves, gastropods (*n* = 6)	104 (98)	19.1 (16)	1.9 (3)	437 (464)	77.5 (227)	4.0 (8)	4.4 (17)	56.3 (180)	9.4 (15)
Cephalopods (*n* = 2)	78.5	17.2	1.1	285	14.5	0.8	1.4	21.5	10.2
Echinoids (*n* = 1)	91.0	8.2	6.5	147	50.0	0.9	0.4	tr	0.0
Sea cucumber (*n* = 1)	52.0	12.8	0.1	716	87.0	1.2	0.2	tr	2.3
Turtle (*n* = 2)	73	16	1.0	129	100	1	1.3	5	1.1
Seaweed (*n* = 2)	9	0.6	0.3	810	56	8	tr	50	0
Canned Fish (mixed fish, oil, brine, other) (*n* = 12)	177 (139)	21.8 (14.7)	9.5 (11.1)	937 (5310)	154 (399)	1.5 (1.7)	1.2 (2.4)	16.8 (37)	3.3 (7.6)

* ‘n’ represents number of species within an aggregated group; mean group values are displayed—the range between the minimum and maximum values in parentheses for groups with three or more species; see supplementary material Table S1 for individual species within groups; ‘tr’ indicates trace amounts detected.

**Table 2:** Mean daily per capita apparent consumption of aquatic foods (edible portion in grams ± standard error) in Vanuatu (2010) and Solomon Islands (2011–12), by national, rural and urban.

	Solomon Islands			Vanuatu		
Aquatic Food Group	National	Rural	Urban	National	Rural	Urban
Pelagic fish	53.2 (1.51)	56.1 (1.92)	45.8 (2.08)	14.4 (0.63)	18.0 (0.82)	4.4 (0.49)
Reef fish	97.5 (2.33)	116.1 (3.07)	50.9 (2.30)	7.2 (0.49)	7.6 (0.58)	6.1 (0.88)
Canned fish	13.5 (0.22)	9.2 (0.19)	24.3 (0.49)	14.1 (0.30)	13.3 (0.30)	16.3 (0.68)
Shellfish	30.7 (1.26)	39.0 (1.67)	9.7 (1.04)	1.6 (0.08)	2.0 (0.11)	0.44 (0.05)
Mixed fresh/frozen fish	6.3 (0.60)	8.3 (0.82)	1.2 (0.38)	1.4 (0.11)	1.7 (0.14)	0.58 (0.16)
**Aquatic food (total)**	201.2 (3.45)	228.8 (4.52)	131.8 (3.70)	38.8 (0.99)	42.6 (1.23)	27.9 (1.40)

## Supplementary Materials

The following are available online at https://www.mdpi.com/2072-6643/12/12/3705/s1, Table S1: Nutritional composition of aquatic foods consumed in the Pacific.
