# Supplementary material for: Aquatic Foods and Nutrition in the Pacific
Source: Nutrients. 2020 Nov 30;12(12):3705. doi: 10.3390/nu12123705 (PMC7761396; doi:10.3390/nu12123705)
Supplement: Supplementary file 1 [file nutrients-12-03705-s001.pdf]

Table S1. Nutritional composition of aquatic foods consumed in the Pacific. Nutrient values are based on 100g edible portions. Mean value displayed. Nutrient composition data from the PNDB.

	Food description in the HIES	Food description in the PNDB	Scientific name	Energy (kcal)	Protein (g)	Fat (g)	Sodium (mg)	Calcium (mg)	Iron (mg)	Zinc (mg)	Vitamin A (µg RAE)	Vitamin B12 (µg)
Tuna	Tuna, albacore	Tuna, albacore, flesh, raw	*Thunnus alalunga*	149.0	26.4	4.7	35.0	9.0	1.0	0.5	47.0	0.7
	Tuna, skip jack	Tuna, skip jack, flesh, raw	*Katsuwonus pelamis*	179.0	25.6	8.2	31.0	3.0	1.7	0.5	78.0	0.7
	Tuna, yellow fin, big eye	Tuna, yellow fin, flesh, raw	*Thunnus albacares*	102	24.4	0.5	45.0	4.0	0.8	0.4	18.0	2.1
	Tuna, not further specified	Tuna, flesh, composite, raw		171	23.9	8.3	42.0	6.0	1.3	0.6	76.0	1.0
Small pelagic	Sardine	Sardine, Australian, whole, raw	*Sardinops sagax*	105.0	19.7	2.9	665.0	725.0	4.0	3.1	106.0	8.3
Large pelagic	Mackerel, Spanish, "Walu"	Mackerel, Spanish, "Walu", raw	*Scombermorus cavalla*	112.0	20.9	3.1	137.0	7.0	0.5	0.6	17.0	0.6
	Fish, pelagic/ocean, not further specified	Fish, pelagic/ocean, composite, raw		149.0	21.0	7.2	83.0	21.0	1.1	0.7	45.0	1.3
Demersal/reef fish	Sweetlip, painted	Sweetlip, painted, raw	*Diagramma pictum*	83.0	19.8	0.2	84.0	39.0	0.4	0.6	29.0	2.1
	Fish, reef, not further specified	Fish, reef, composite, raw		109.0	19.5	3.3	73.0	19.0	0.7	0.6	31.0	2.3
	Snapper	Snapper, flesh, raw	*Pagrus auratus*	111.0	20.1	3.2	78.0	18.0	0.6	0.5	31.0	0.7

Elasmo-branchs	Shark	Shark, flesh, composite, raw	*Isurus oxyrinchus*	95.0	20.9	1.2	108.0	12.0	1.3	0.5	12.0	0.7
	Stingray	Stingray, raw		100.0	24.2	0.3	87.0	10.0	0.7	0.4	12.0	1.4
Canned Fish (mixed fish, oil, brine, other)	Tuna, canned in oil	Tuna, canned in oil, drained		221.0	24.4	13.7	441.0	7.0	0.8	0.9	20.0	1.3
	Tuna, canned in tomato	Tuna, canned in tomato		111.0	15.1	4.2	430.0	13.0	1.6	0.8	27.0	1.4
	Tuna canned, not further specified	Tuna canned, composite		153.0	21.9	6.8	357.0	8.0	1.2	0.8	16.0	1.3
	Tuna, canned in brine	Tuna, canned in brine, drained		128.0	26.1	2.6	200.0	5.0	1.2	0.8	0.0	1.1
	Herring, canned, not further specified	Herring, Atlantic, canned in brine, pickled, drained		250.0	14.2	18.0	870.0	77.0	1.2	0.5	17.0	4.3
	Mackerel, canned, not further specified	Mackerel, canned, composite		182.0	19.2	11.2	360.0	287.0	1.5	1.3	11.0	8.7
	Sardines, canned, not further specified	Sardines, canned, composite		181.0	19.9	10.9	399.0	404.0	2.0	1.9	37.0	4.1
	Anchovy, canned, not further specified	Anchovy, canned in oil, drained		203.0	28.9	9.7	5480.0	167.0	2.5	2.9	4.0	0.9
	Salmon, canned, not further specified	Salmon, canned, unflavoured,		158.0	22.2	7.7	170.0	249.0	0.9	1.0	21.0	1.3

		drained, composite										
	Fish, canned in oil, not further specified	Fish, composite, canned in oil, drained		203.0	23.7	12.1	1380.0	234.0	1.6	1.6	24.0	2.7
	Fish, canned in brine, not further specified	Fish, composite, canned in brine, drained		158.0	22.7	7.4	308.0	184.0	1.5	1.2	8.0	7.4
	Fish, canned, not further specified	Fish, canned, composite, drained		181.0	23.2	9.7	846.0	209.0	1.5	1.4	16.0	5.1
Prawns/ shrimp	Prawn/shrimp, not further specified	Prawn/shrimp, flesh, composite, raw		92.0	20.4	1.2	249.0	89.0	1.6	1.3	27.0	1.5
Crabs/ crayfish	Crab, coconut	Crab, mud, fresh, raw	*Birgus latro*	71.0	14.0	1.7	305.0	192.0	1.7	3.3	12.0	2.1
	Crab, land	Crab, mud, fresh, raw		71.0	14.0	1.7	305.0	192.0	1.7	3.3	12.0	2.1
	Crab, mud	Crab, mud, fresh, raw	*Scylla serrata*	71.0	14.0	1.7	305.0	192.0	1.7	3.3	12.0	2.1
	Crab, not further specified	Crab, flesh, composite, raw	*species belonging to the Portunidae family*	78.0	17.1	0.9	362.0	88.0	0.6	3.5	33.0	6.0
	Crayfish / lobster, not further specified	Crayfish / lobster, composite, raw		89.0	19.2	1.0	118.0	53.0	0.9	3.9	12.0	2.2
Bivalves & gastropods	Mussels	Mussels, raw	*Mytilus coruscus*	115.0	20.1	2.8	479.0	64.0	3.8	2.3	189.0	16.0

	Scallop	Scallop, raw	*Dolabella auricularia*	73.0	13.8	1.2	250.0	49.0	1.0	0.8	13.0	1.3
	Oyster	Oyster, Pacific, flesh, raw	*Crassostrea gigas*	65.0	10.8	2.2	541.0	229.0	4.4	18.0	27.0	15.0
	Sici-shell, meat	Sici-shell, meat, raw	*Polinicies aemingiana*	115.0	26.6	0.9	714.0	2.0	2.3	1.5	9.0	15.0
	Sea snail	Paua, raw	*Haliotis spp.*	95.0	20.6	1.0	356.0	30.0	9.3	0.7	60.0	8.0
	Trochus	Sea snail		163.0	22.7	3.5	279.0	91.0	3.2	2.9	40.0	0.8
Cephalopods	Octopus	Octopus, raw	*Octopus vulgaris*	72.0	16.4	0.7	280.0	16.0	0.6	1.6	43.0	19.0
	Squid, not further specified	Squid, composite, raw		85.0	17.9	1.5	290.0	13.0	1.0	1.2	0.0	1.3
Sea urchin	Sea urchin	Sea urchin, raw	*Tripneustes gratilla*	91.0	8.2	6.5	147.0	50.0	0.9	0.4	tr	0.0
Sea cucmber	Beche de mar/sea cucumber	Sea cucumber, edible muscle	*Stichopus japonicus*	52.0	12.8	0.1	716.0	87.0	1.2	0.2	tr	2.3
Seaweed	Seaweed	Seaweed, fresh, raw		9.0	0.6	0.3	810.0	56.0	8.0	tr	50.0	0.0
Turtle	Turtle	Turtle, raw	*Chelonia mydas*	73.0	16.0	1.0	129.0	100.0	1.0	1.3	5.0	1.1

Figure 3 list of countries:

Fiji, New Caledonia, Solomon Islands, Vanuatu, Federated State of Micronesia, Kiribati, Marshall Islands, Nauru, Palau, Cook Islands, French Polynesia, Niue, Samoa, Tokelau, Tonga, Tuvalu, Wallis and Futuna Islands.

Papua New Guinea was excluded because it is more agriculture focused and has a human population size which exceeds all other PICTs combined, and would therefore skew interpretation.
